# Efficacy of Noninvasive Stellate Ganglion Blockade Performed Using Physical Agent Modalities in Patients with Sympathetic Hyperactivity-Associated Disorders: A Systematic Review and Meta-Analysis

**DOI:** 10.1371/journal.pone.0167476

**Published:** 2016-12-02

**Authors:** Chun-De Liao, Jau-Yih Tsauo, Tsan-Hon Liou, Hung-Chou Chen, Chi-Lun Rau

**Affiliations:** 1 School and Graduate Institute of Physical Therapy, College of Medicine, National Taiwan University, Taipei, Taiwan; 2 Department of Physical Medicine and Rehabilitation, Shuang Ho Hospital, Taipei Medical University, Taipei, Taiwan; 3 Center for Evidence-Based Health Care, Shuang Ho Hospital, Taipei Medical University, Taipei, Taiwan; 4 Graduate Institute of Injury Prevention and Control, Taipei Medical University, Taipei, Taiwan; 5 Department of Physical Medicine and Rehabilitation, School of Medicine, College of Medicine, Taipei Medical University, Taipei, Taiwan; University of Bern, SWITZERLAND

## Abstract

**Background:**

Stellate ganglion blockade (SGB) is mainly used to relieve symptoms of neuropathic pain in conditions such as complex regional pain syndrome and has several potential complications. Noninvasive SGB performed using physical agent modalities (PAMs), such as light irradiation and electrical stimulation, can be clinically used as an alternative to conventional invasive SGB. However, its application protocols vary and its clinical efficacy remains controversial. This study investigated the use of noninvasive SGB for managing neuropathic pain or other disorders associated with sympathetic hyperactivity.

**Materials and Methods:**

We performed a comprehensive search of the following online databases: Medline, PubMed, Excerpta Medica Database, Cochrane Library Database, Ovid MEDLINE, Europe PubMed Central, EBSCOhost Research Databases, CINAHL, ProQuest Research Library, Physiotherapy Evidence Database, WorldWideScience, BIOSIS, and Google Scholar. We identified and included quasi-randomized or randomized controlled trials reporting the efficacy of SGB performed using therapeutic ultrasound, transcutaneous electrical nerve stimulation, light irradiation using low-level laser therapy, or xenon light or linearly polarized near-infrared light irradiation near or over the stellate ganglion region in treating complex regional pain syndrome or disorders requiring sympatholytic management. The included articles were subjected to a meta-analysis and risk of bias assessment.

**Results:**

Nine randomized and four quasi-randomized controlled trials were included. Eleven trials had good methodological quality with a Physiotherapy Evidence Database (PEDro) score of ≥6, whereas the remaining two trials had a PEDro score of <6. The meta-analysis results revealed that the efficacy of noninvasive SGB on 100-mm visual analog pain score is higher than that of a placebo or active control (weighted mean difference, −21.59 mm; 95% CI, −34.25, −8.94; *p* = 0.0008).

**Conclusions:**

Noninvasive SGB performed using PAMs effectively relieves pain of various etiologies, making it a valuable addition to the contemporary pain management armamentarium. However, this evidence is limited by the potential risk of bias.

## Introduction

The prevalence of chronic pain is 20%–30% in the general population, and approximately one-fifth of people who complain of chronic pain are believed to predominantly experience neuropathic pain. Neuropathic pain syndromes are particularly distressful chronic pain syndromes affecting 2%–8% of the general population and their quality of life. Neuropathic pain syndromes are clinically characterized by spontaneous, stimulus-independent, persistent pain. Moreover, a sympathetically maintained component is a common feature of these syndromes, along with multiple α-adrenergic sensitization-associated subsets dependent on activity in the sympathetic nervous system [1].

Sympathetic overflow or hyperactivity is a common clinical feature of neuropathic pain syndromes such as complex regional pain syndrome (CRPS) type I [2–4], fibromyalgia [5–7], and trigeminal neuralgia [8]. Pain can be enhanced or maintained by functional abnormalities of the sympathetic nervous system, including functional sympathetic afferent coupling and increased adrenergic receptor expression at the peripheral terminals of nociceptive afferent nerve fibers, resulting in the release of neuropeptides [substance P and calcitonin gene-related peptide (CGRP)] from peptidergic unmyelinated fibers [4, 9–11]. In addition to pain, excessive sympathetic outflow originating from small-fiber neuropathy (e.g., CRPS and reflexive sympathetic dystrophy) leads to changes in sympathetic vasoconstrictor activity and sudomotor dysfunction, which might be clinically represented as skin temperature and/or color changes, swelling, edema, or hyperhidrosis (i.e., spontaneous, thermoregulatory, and sudomotor axon reflex sweating) in the affected extremity [2, 3]. Furthermore, vasomotor abnormalities or hyperhidrosis responding to neurogenic inflammation alter the concentration of peripheral neuropeptides in the affected tissue, such as the antidromic release of the vasodilated neuromediator CGRP or the vasoconstrictive neuropeptide endothelin-1 by the endings of afferent polymodal C fibers and efferent sympathetic fibers that critically regulate vasomotor and tropic efferent functions [4, 10, 11]. Therefore, the modulation of sympathetic activity by using a sympathetic inhibitor or a local sympathetic ganglion blockade may affect the pain course in patients with chronic pain and hyperalgesia that are suspected to be sympathetically maintained [12].

Stellate ganglion blockade (SGB), a local anesthetic blockade of the sympathetic ganglia, is used in clinical practice to manage various vascular disorders and pain conditions including upper extremity, nuchal, cephalic, and atypical facial pain. SGB has been advocated as an early intervention for achieving sympatholysis through the blockade of efferent sympathetic nerves [13–16]; however, the efficacy and safety of sympathetic blockades remain unclear [17]. Moreover, the success of conventional SGB depends on the skill through which the invasive technique is applied. In addition, the following serious complications can occur during or after the application of the anterior paratracheal technique: local muscle injury and scarring caused by repeated injections at the same point [18]; convulsions caused by intraarterial injections (incidence: 1.7 per 1000 blockades) [19, 20]; esophageal puncture [21]; retropharyngeal hematoma or recurrent laryngeal or phrenic nerve blockade resulting in fatal respiratory arrest [22–24]; locked-in syndrome [25, 26]; pneumothorax [23]; sinus arrest [27]; serious cervical hematoma [28]; and severe arterial hypotension [29]. Moreover, repeated application of the technique can cause recurrent paralysis of the involved nerves.

The sympathetic nerves are of particular interest in pain treatment. Therefore, numerous noninvasive approaches for SGB employing physical agent modalities (PAMs) have been developed as alternatives to the conventional invasive anesthetic technique, including therapeutic ultrasound (US), transcutaneous electrical nerve stimulation (TENS), light irradiation using low-level laser therapy (LLLT), and xenon light and linearly polarized near-infrared (LPNIR) light irradiation near or over the stellate ganglion region. In addition, noninvasive SGB can be safely and conveniently performed in clinical practice, particularly in patients declining injections, having a high bleeding tendency, undergoing anticoagulant therapy, or having contraindications for nerve blockade, such as those with hemophilia [30–34]. In patients with neuropathic pain syndromes, the effects of SGB performed using light irradiation were similar to those of conventional intensive SGB, including improved blood flow through vasodilation and reduced pain by direct blockade of the afferent nociceptive signals traveling through sympathetic pathways [31, 33, 35–38]. Moreover, the effects of SGB performed using TENS [39–41] and therapeutic US [41, 42] were similar. Compared with the conventional nerve blockade technique, noninvasive SGB is free from potential complications such as infection, bleeding, potential nerve damage, and other adverse events that may be caused by an injective or a puncture injury following repeated applications [19–29]. Moreover, noninvasive SGB can be conveniently performed in clinical practice even in the absence of an anesthesiologist and is well tolerated by patients without any thermal injury or with few adverse effects [31, 39, 40, 43–56], regardless of the application modality.

Noninvasive SGB can provide clinically effective pain relief, improve peripheral vasomotor and sudomotor dysfunction and abnormal heart rate variability (HRV), and maintain homeostasis in patients with neuropathic pain syndromes such as CRPS [40, 41, 43, 44, 46, 53, 57], fibromyalgia [33], glossodynia [52], burning mouth syndrome [31, 36, 58], postherpetic neuralgia [49, 59, 60], trigeminal neuralgia [61], and thalamic pain [55] as well as in those with other disorders such as Bell’s palsy [50, 51, 60], musculoskeletal pain [38], postoperative sensory disturbance [62], Raynaud’s phenomenon [63], glaucoma [64], and sudden deafness [65]. In addition, noninvasive SGB can alleviate conditions associated with hypersympathetic tone [35, 45, 47, 64–68] and physiological changes associated with suppressed sympathetic activities in healthy adults [34, 37, 42, 56, 69–75]. Because of the heterogeneity of treatment protocols and study designs, careful interpretation of results and drawing of conclusions regarding the short- and long-term efficacy of noninvasive SGB are necessary. In addition, because each PAM has various applications, providing prompt and definite guidance to clinicians performing SGB as the primary procedure in clinical practice becomes difficult. Therefore, a review on the efficacy of noninvasive SGB reported in studies using various methodologies is urgently required.

Despite the increased use of PAMs in pain management, the effectiveness of their application in sympathetic blockade has only been sporadically examined. Most studies examining this topic have used a nonrandomized experimental design or case series [31, 33, 36, 45, 64, 76–81]. Several reviews and meta-analyses of the effectiveness of PAMs in pain management have been published for assisting clinicians in making decisions [82–87]. However, few systematic meta-analyses have provided adequate evidence on the efficacy of noninvasive SGB performed using PAMs.

We conducted a systematic review and meta-analysis to determine the effectiveness of noninvasive SGB in managing neuropathic pain and disorders associated with sympathetic nervous system dysfunction.

## Materials and Methods

### Design

This study was approved by the Ethical Review Board of Taipei Medical University (protocol number: N201602100) and conducted in accordance with the guidelines recommended by the Preferred Reporting Items for Systematic Reviews and Meta-Analysis [88]. We conducted a comprehensive electronic database search. Original research articles on the clinical efficacy of SGB performed using noninvasive PAMs for pain management published between January 1950 and December 2015 were aggregated and coded. The articles were obtained from the following online databases: Physiotherapy Evidence Database, Medline, PubMed, Excerpta Medica Database, Cochrane Library Database, Ovid MEDLINE, Europe PubMed Central, EBSCOhost Research Databases, ProQuest Research Library, WorldWideScience, BIOSIS, and Google Scholar. Secondary sources were papers cited by the articles retrieved from these databases and articles published in journals that were not available in these databases. The search was restricted to published or in-press articles on human studies, without language restriction. Non-English language papers were translated to English. In addition, we consulted anesthesiology and neurology experts to conduct a systematic review and meta-analysis of noninvasive SGB for pain management. Two reviewers (CDL and CLR) independently searched articles, screened studies, and extracted data in a blinded manner with adequate reliability. Any disagreements between the reviewers were resolved through consensus with other team members (HCC and THL) acting as arbiters.

### Search strategy

We used the following search terms to identify articles on neuropathic pain and associated conditions: “chronic pain/syndrome,” “neuropathic pain/syndrome,” “complex regional pain syndrome type I/type II,” “reflex sympathetic dystrophy,” “fibromyalgia,” “glossodynia,” “burning mouth syndrome,” “postherpetic/trigeminal neuralgia,” “neuralgia,” “thalamic pain,” “Bell’s/facial palsy,” “musculoskeletal pain,” “postoperative sensory disturbance,” “post-traumatic pain disorders,” “Raynaud’s phenomenon/disease/syndrome,” “sympathetic dysfunction/hyperactivity,” “sympathetically maintained pain syndrome,” “CRPS,” and “RSD.” Furthermore, search terms used for SGB were “stellate ganglion,” “stellate ganglion block/blockade,” “sympathetic (ganglion),” and “sympathetic (ganglion) block/blockade.” On the basis of previous studies [83, 84], we used the following search terms for light therapy: “laser therapy,” “low-energy photon therapy,” “low output laser,” “low-level laser therapy,” “LLLT,” “LASER,” “photobiomodulation,” “phototherapy,” “light therapy/(ir)radiation,” “narrow-band light therapy,” and “linear(ly) polarized infrared light.” The search terms used for therapeutic US were “ultrasound/ultrasonic/US therapy” and “therapeutic ultrasound.” The terms used for TENS were “transcutaneous electrical nerve stimulation,” “electric(al)/electricity/electrotherapy/stimulation,” “transdermal electroimpulses,” “low-level transcutaneous electrical stimulation,” “diadynamotherapy,” “diadynamic therapy,” “diadynamic current,” “electroacupuncture,” “electroanaesthesia,” and “external noninvasive peripheral nerve stimulation.” Other common search terms for noninvasive interventions included “electrophysical agent/modality,” “biostimulation,” and “neuromodulation.”

### Study selection criteria

Article were included if they fulfilled the following criteria [89]: (1) the article was published or in press in a peer-reviewed, scientific journal; (2) it was published between January 1950 and December 2015; (3) it reported an in vivo human trial only; (4) the trial design was randomized or quasi-randomized and controlled, and the trial concerned sympathetic blockade using noninvasive SGB for patients with neuropathic pain disorders with or without sympathetic hyperactivity [90]; (5) the trial was conducted using an electrophysical modality that delivered US, light irradiation, or electrostimulation to or on the area near the stellate ganglion on either the right or left side; (6) control groups were administered a placebo using sham irradiation or stimulation or they underwent active treatment (e.g., exercise and other physical therapeutic modalities); (7) the trial included a cointervention, such as pharmacological and conventional invasive SGB, or other physical therapies in both placebo and noninvasive SGB groups; (8) pain was measured using a quantifiable scale or outcome, such as the visual analog scale (VAS), and (9) the following application parameters could be extracted: source of stimulation, wavelength, power, power density, number and duration of treatment sessions, frequency of treatment, dose (intensity), side of the area treated, and mode of treatment (continuous or pulse mode for therapeutic US and monophasic or biphasic mode for TENS).

Articles on studies using animal models, case reports, and case series were excluded. In addition, non-English articles that could not be translated into English were excluded.

### Data extraction

We developed a data extraction sheet for the included studies and refined it accordingly [91]. An author (CDL) extracted the relevant data from the included studies, and another author (CLR) reviewed the extracted data. Any disagreement between the two authors was resolved through consensus. A third author (THL) was consulted if the disagreement persisted.

### Outcome measures

The effects of noninvasive SGB on primary outcomes including pain intensity, sympathetic skin response, peripheral blood flow or vascular conductance, and peripheral skin temperature were calculated as weighted mean differences (WMDs) or standard mean differences (SMDs) versus the placebo or active control. In addition, secondary outcomes including functional mobility and disability were calculated as SMDs versus the placebo or active control.

### Assessment of bias risk and methodological quality

Quality assessment was performed using the Physiotherapy Evidence Database (PEDro) quality scale, a valid measure of the methodological quality of clinical trials [92], to assess the risk of bias. The PEDro scores of the following 10 items were determined: random allocation, concealed allocation, similarity at the baseline, subject blinding, therapist blinding, assessor blinding, >85% follow-up for at least one key outcome, intention-to-treat analysis, between-group statistical comparison for at least one key outcome, and point and variability measures for at least one key outcome. Each item was scored as 1 when a criterion was clearly satisfied or 0 when the criterion was unclear or absent; the final sum of the scores (0–10) was obtained by summing the scores for all 10 items. The methodological quality of all included studies was independently and blindly assessed by two researchers (CDL and HCC) according to the PEDro classification scale. If any item of the assessed study had different graded scores, it was further ranked by a third assessor (THL). The interrater reliability measured using the generalized kappa statistic is between 0.40 and 0.75 for the PEDro scale [93]. The intraclass correlation coefficient associated with the cumulative PEDro score is 0.91 [95% confidence interval (CI): 0.84–0.952] for nonpharmacological studies [94]. The methodological quality of the included studies was rated from excellent to poor on the basis of the PEDro score: 9–10, excellent; 6–8, good; 4–5, fair; and <4, poor.

We examined adverse events when reported even if they were not specified a priori. The duration of follow-up was assessed and defined as immediate (<1 day), short term (<1 month), medium term (1–6 months), and long term (>6 months) [89].

### Statistical analysis

We separately computed the effect size of each study for the primary and secondary outcome measures after noninvasive SGB. The primary outcomes were defined as pooled estimates of the mean difference in changes between the mean of the treatment and placebo (or active control) groups, weighted by the inverse of the standard deviation (SD) for every included study. If the exact variance of paired differences was not derivable, it was imputed by assuming a correlation coefficient of 0.8 between the baseline and posttreatment pain scores [95, 96]. If data were reported as a median (range), they were recalculated algebraically from the trial data for imputing the sample mean and SD [97]. The odds ratio with a 95% CI was calculated for dichotomous outcomes. For the secondary outcomes, the effect size was defined as an SMD, which was a combined outcome measure without units.

Fixed-effects or random-effects models were used depending on the presence of heterogeneity. Statistical heterogeneity was assessed using the *I*^2^ statistics for significance (*p* < 0.05) and χ^2^ and *F* values of >50% [98]. The fixed-effects model was used when significant heterogeneity was absent (*p* > 0.05), whereas the random-effects model was used when heterogeneity was significant (*p* < 0.05).

Subgroup analysis was performed on the basis of the therapy type and methodological quality of the included studies. Potential publication bias was investigated through visual inspection of a funnel plot for exploring possible reporting bias [99] and was assessed using Egger’s regression asymmetry test [100], by using SPSS (Version 17.0; IBM, Armonk, NY, USA). A value of *p* < 0.05 was considered to be statistically significant. All analyses were conducted using RevMan 5.3 (The Nordic Cochrane Centre, Copenhagen, Denmark).

## Results

### Selection process

**Fig 1:**
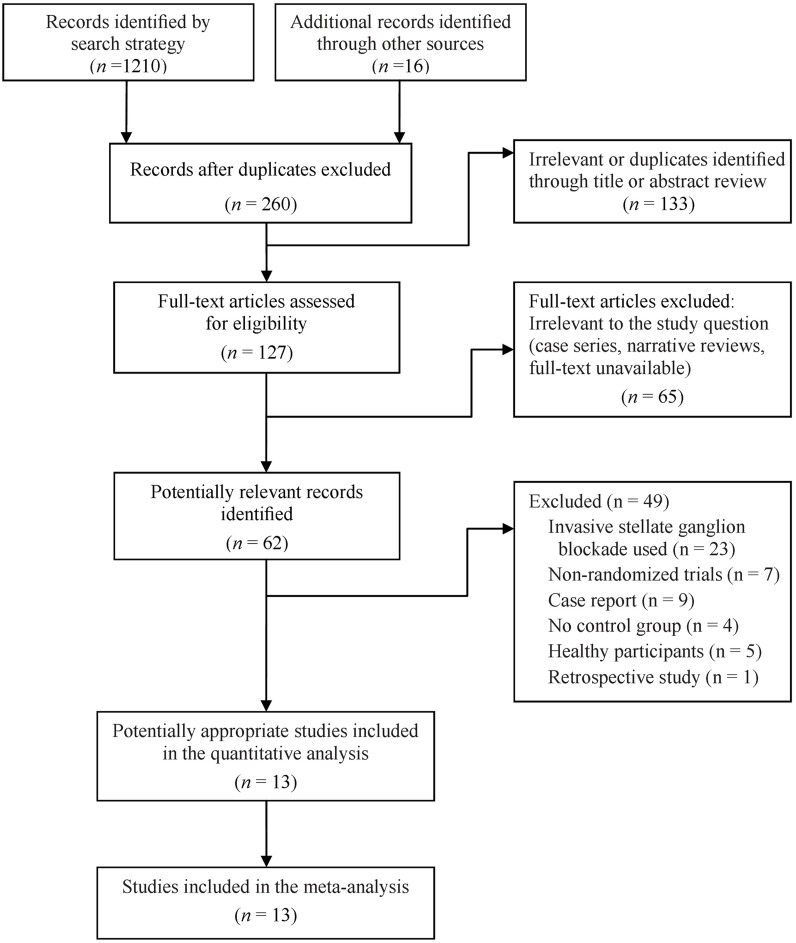
Flow chart of study selection.

Fig 1 illustrates the flow chart of the selection process. The final sample consisted of nine randomized placebo- or active-controlled [40, 43–50] and four quasi-randomized [51–54] trials published between 1994 and 2014 with a total sample size of 440 patients.

### Study characteristics

Table 1 lists the demographic data and study characteristics of the included trials. Noninvasive SGB was performed using therapeutic US, TENS, and light irradiation in two [43, 44], four [40, 45, 47, 48], and seven [46, 49–54] trials, respectively. The applied parameters of modalities used for SGB and treatment protocols are summarized in Table 2.

**Table 1:** Demographics and study characteristics of the included trials.

Study	Age (y/r)	Sex F/M	No. per group	Design	Condition treated	Side treated	Groups	Cointervention	Total treatment sessions (duration)	Measurement time points	Outcome measures
	Mean (SD)										
Askin (2014) [43]	45.5 (13.3)	19/21	13	DB	CRPS (type I)	NA	Gr 1: SG-US (0.5 wt/cm^2^)	Pharmacological medication	20 (6 weeks)	Pretest	VAS
	46.0 (13.3)		13	RCT			Gr 2: SG-US (3 wt/cm^2^)	TENS		Posttest	DASH
	44.8 (13.6)		14				Gr 3: Placebo	Contrast bath			SSR
								Exercise			
Aydemir (2006) [44]	21.9 (1.05)	NA	9	RCT	CRPS (type I)	R/L/Bil	Gr 1: SGB + sham SG-US	Exercise	21	Pretest	VAS
	21.4 (0.73)		9				Gr 2: SG-US + sham SGB	Contrast bath		Posttest	Edema
	21.1 (0.38)		7				Gr 3: Placebo	TENS		Follow-up: 1 month	Grip strength
								Pneumatic compression			Keitel score
Cipriano (2014) [47]	62.0 (4.0)	18/20	20	DB	CAD	NA	Gr 1: SG-TENS	Pharmacological medication	20 (1 week)	Pretest	VAS
	66.0 (3.0)		18	RCT			Gr 2: Placebo			Posttest	Opioid usage
											BP
											Femoral blood flow
											6MWT
Barker (2007) [45]	65.3 (18.3)	32/21	53	RCT	Posttraumatic vasoconstriction and hypothermia	R/L	Gr 1: SG-TENS treated limb	NA	1	Pretest	Pulse oximeter dropout alerts
			53				Gr 2: Opposite-side control			Posttest	(alarm duration and frequency)
											Difference between core and skin temperature
Bolel (2006) [40]	44.6 (16.4)	12/18	15	RCT	RSD	R/L	Gr 1: SG-TENS	Pharmacological medication	1	Pretest	SSR
	41.1 (20.8)		15				Gr 2: Control*	Exercise		Posttest	
								Physical therapy agents			
								(hot/cold pack, whirl-pool, TENS)			
Fassoulaki (1994) [48]	45.8 (8.0)	NA	19	RCT	Hysterectomy operation	NA	Gr 1: SG-TENS	NA	1	Preoperation	BP
	44.6 (10.7)		19				Gr 2: Placebo			Intraoperation	HR
										Postoperation: 2–8 h	
Nakase (2004) [52]	66.0 (9.3)	49/15	37	Quasi-randomized	Glossodynia	R/L/Bil	Gr 1: SGL	NA	16 (4 weeks)	Pretest	VAS
	64.9 (12.4)		19	active-controlled			Gr 2: Gargle medication			Posttest	Tongue temperature
	63.1 (16.0)		8				Gr 3: SGL to healthy				Tongue blood flow
Basford (2003) [46]	45.8 (12.3)	5/1	6	DB	CRPS (type I)	R	Tr 1: SGL	NA	1	Pretest	VAS
				Randomized crossover			Tr 2: Placebo			Posttest, 30 min	HRV
				placebo-controlled						Follow-up: 1–2 weeks	HR
											Skin temperature
											Digital blood flow
Wee (2001) [53]	59.0 (1.4)	NA	20	Quasi-randomized Crossover	RSD	R/L	Gr 1: SGL limb	NA	30 (6 weeks)	Pretest	VAS
	59.0 (1.4)		20				Gr 2: Control limb			Posttest	Finger circumference
											Skin temperature
Kudoh (1998) [50]	44.5 (1.6)	27/23	25	RCT	Facial palsy	R/L	Gr 1: SGL	Pharmacological medication	2–4 session/week (3 months)	Pretest	Electroneurography
	43.7 (2.3)		25				Gr 2: Control*			Mid-time point:	Paralysis score
										1, 2, & 3 months	
Hashimoto (1997) [49]	66.3 (5.4)	2/6	8	DB	PHN	R/L	Tr 1: SGL (150W)	NA	1	Pretest	VAS
				Randomized crossover			Tr 2: SGL (60W)			Posttest	Skin temperature
				placebo-controlled			Tr 3: Placebo			Follow-up 5–30 min	
Yamada (1995) [54]	43.6 (12.5)	13/11	7	Quasi-randomized	Hunt’s syndrome II	NA	Gr 1: SGL	NA	21–66 (5–12 weeks)	Pretest	Paralysis score
	45,1 (14.0)		7		Bell’s palsy		Gr 2: SGL + PM			Mid-time point: 2 weeks	
	43.2 (10.9)		10				Gr 3: PM only			Follow-up 5–12 weeks	
Murakami (1993) [51]	45.3 (4.1)	52	11	Quasi-randomized	Facial palsy	NA	Gr 1: SGL	Pharmacological medication	NA	Posttest 1, 7, 14, 21, 30 & >30 days	Paralysis score
	43,5 (4.1)		15				Gr 2: SGB				
	41.8 (4.7)		26				Gr 3: SGL + SGB				

DB = double blind; RCT = randomized control trial; NA = not available; R = right; L = left; Bil = bilateral; Gr = group; Tr = treatment; CRPS = complex regional pain syndrome; CAD = coronary artery disease; RSD = reflex sympathetic dystrophy; PHN = postherpetic neuralgia; SG-US = ultrasound therapy to the stellate ganglion; SGB = stellate ganglion blockade; TENS = transcutaneous electrical nerve stimulation; SGL = Light irradiation to the stellate ganglion; VAS = visual analog scale; DASH = Disability of the Arm, Shoulder, and Hand scale; SSR = sympathetic skin response; BP = blood pressure; 6MWK = 6-min walk test; HR = heart rate; HRV = heart rate variability

* no intervention to the stellate ganglion

**Table 2:** Source of stimulation, wavelength, power, power density, and energy.

Study	Source of stimulation^a^	Wavelength/ Frequency	Application parameters	Duration (min)	Power (W)	Power Density (W/cm^2^)	Energy
Askin (2014)[43]	Therapeutic US	1 MHz	1-cm^2^ heading applicator	5		0.5	300 J/cm^2^
			Pulse pattern, 1:4			3.0	180 J/cm^2^
Aydemir (2006)[44]	Therapeutic US	1 MHz	1-cm^2^ heading applicator	5		3.0	180 J/cm^2^
Cipriano (2014)[47]	TENS	40–80 Hz	Pulse duration: 150–200 μs	30			
			Pain-free stimulation intensity (mA)				
Barker (2007)[45]	TENS	100 Hz	Pulse duration: 200 μs	NA^b^			
			Intensity: 15 mA				
Bolel (2006)[40]	Diadynamic current	50–100 Hz	NA	NA			
Fassoulaki (1994)[48]	TENS	40 Hz	Intensity 12–29 mA	600			
			(mean ± SD, 18 ± 4 mA)				
Nakase (2004)[52]	LPNIR light	600–1600 nm	Duty cycle (on/off ratio), 1 s/2 s	10	0.97	NA	194.8 J/cm^2^
Basford (2003)[46]	LPNIR light	600–1600 nm	Duty cycle (on/off ratio), 1 s/4 s	8	0.92	0.6	88.3 J
							287 J/cm^2^
Wee (2001)[53]	Helium-neon laser	NA	Duty cycle (on/off ratio), 1 s/5 s	20	1.44	NA	18 J
Kudoh (1998)[50]	LPNIR light	600–1600 nm	Duty cycle (on/off ratio), 1 s/2 s	10	1.44	NA	289.2 J/cm^2^
Hashimoto (1997)[49]	GaAlAs semiconductor laser	830 nm		3	0.15	NA	27 J
					0.06		10.8 J
Yamada (1995)[54]	GaAlAs semiconductor laser	830 nm		5	0.15	0.21	18J
							63.7 J/cm^2^
Murakami (1993)[51]	GaAlAs semiconductor laser	830 nm		2–3	0.06	NA	NA

^a^ US = ultrasound; TENS = transcutaneous electric nerve stimulation; LPNIR = linear polarized near infrared; NA = not available

^b^ This was applied during transportation to the hospital.

Of the 13 trials, six reported co-interventions, with one using physical therapy [44], three allowing pharmacological medication [47, 50, 51], and two combining physical therapy and medication as a between-group co-intervention [40, 43]. With regard to treated conditions, patients with neuropathic pain disorders, namely CRPS type I [40, 43, 44, 46, 53], glossodynia [52], and postherpetic neuralgia [49], were treated in seven trials; patients with atypical facial palsy [50, 51, 54] were treated in three trials; patients with conditions related to sympathetic cardiovascular changes following a coronary artery bypass graft surgery [47] or a hysterectomy [48] were treated in two trials; and patients with posttraumatic hypothermia-related vasoconstriction were treated in one trial by applying TENS on the area near the stellate ganglion [45].

The immediate analgesic and sympatholytic effects of noninvasive SGB and the short-term follow-up of clinical outcomes within 1 month after the end of the treatment protocol were reported in five trials conducting one SGB session [40, 45, 46, 48, 49] and seven trials performing 6–22 SGB sessions using various protocols within an overall treatment period of 1–12 weeks [43, 44, 47, 50, 52–54]. The medium-term follow-up of clinical outcomes 3 months after the end of the treatment protocol was reported in three trials [50, 51, 54]. None of the included trials reported long-term outcomes.

### Risk of bias in the included studies

The two assessors primarily calculated the same PEDro score for the nine included studies [40, 43–46, 48–50, 54]. The third assessor determined the PEDro score of the remaining four trials [47, 51–53]. The interrater reliability associated with the cumulative PEDro score was acceptable with an intraclass correlation coefficient of 0.98 (95% CI: 0.94–0.99). The methodological quality was high for all the included studies with a median (range) PEDro score of 6 (5–8). The methodological quality of 10 and 3 trials was classified as good and fair, respectively. The individual PEDro scores are listed in Table 3. Of the 13 studies, 9 had random allocation, 5 had concealed allocation, all had similarity at the baseline, 6 incorporated subject blinding, 3 incorporated therapist blinding, 4 incorporated assessor blinding, 9 had adequate follow-up, 10 had intention-to-treat analysis, 11 had between-group comparisons, and all had point estimates and variability.

**Table 3:** Summary of the methodological quality based on the PEDro classification scale.

Study	Overall^a^	EligibilityCriteria^#^	Random allocation	Concealedallocation	Baseline comparable	Subject Blinding	Therapist Blinding	Assessor Blinding	Adequate follow-up	Intention to treat	Between-group comparison	Point estimates & variability
Askin (2014)[43]	7	*X*	*X*	*X*	*X*	*X*		*X*			*X*	*X*
Aydemir (2006)[44]	7	*X*	*X*	*X*	*X*	*X*		*X*	*X*			*X*
Cipriano (2014)[47]	6^¶^	*X*	*X*		*X*	*X*					*X*	*X*
Barker (2007)[45]	7	*X*	*X*	*X*	*X*				*X*	*X*	*X*	*X*
Bolel (2006)[40]	6	*X*	*X*	*X*	*X*					*X*	*X*	*X*
Fassoulaki (1994[48]	8	*X*	*X*		*X*	*X*	*X*	*X*	*X*	*X*	*X*	*X*
Nakase (2004)[52]	5^¶^	*X*			*X*				*X*	*X*	*X*	*X*
Basford (2003)[46]	8	*X*	*X*	*X*	*X*	*X*	*X*	*X*		*X*		*X*
Wee (2001)[53]	5^¶^	*X*			*X*				*X*	*X*	*X*	*X*
Kudoh (1998)[50]	6	*X*	*X*		*X*				*X*	*X*	*X*	*X*
Hashimoto (1997)[49]	8	*X*	*X*		*X*	*X*	*X*		*X*	*X*	*X*	*X*
Yamada (1995)[54]	6	*X*			*X*				*X*	*X*	*X*	*X*
Murakami (1993)[51]	5^¶^	*X*			*X*				*X*	*X*	*X*	*X*
Summary*		13	9	5	13	6	3	4	9	10	11	13

^a^ Points of methodological quality were “X” when a criterion was fulfilled. Methodological quality: 9–10, excellent; 6–8, good; 4–5, fair; <4, poor.

^¶^ The score was determined by a third assessor.

^#^ This item was not used to calculate the total score.

* This was calculated as the number of studies satisfied

### Effect on pain relief

**Fig 2:**
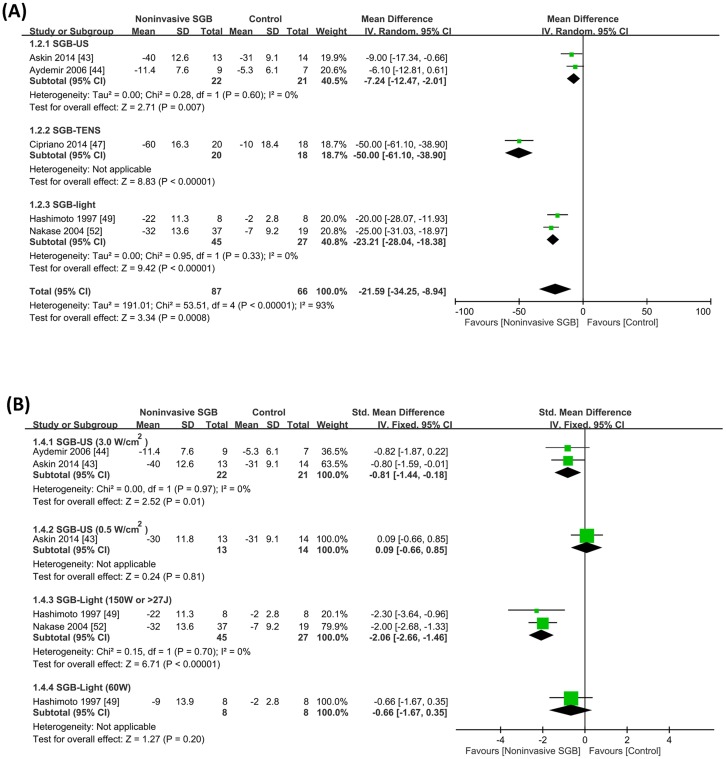
(A) Weighted mean differences in pain reduction on a 100-mm visual analog scale between noninvasive stellate ganglion blockade (SGB) and placebo groups from five controlled trials grouped according to the type of electrophysical modality used. (B) Subgroup analysis of high- and low-dose noninvasive SGB. Trial results plotted on the left-hand side indicate effects favoring noninvasive SGB, and the combined effects are plotted using black diamonds. US = ultrasound; TENS = transcutaneous electrical nerve stimulation.

The five trials [43, 44, 47, 49, 52] determined pain intensity on a VAS. All VAS data were transformed to 0–100-mm continuous data. The analysis of the transformed VAS data revealed that compared with the control group, pain decreased in the SGB group by a WMD of −21.59 mm (95% CI, −34.25, −8.94; *p* = 0.0008), irrespective of the methodological quality used. Moreover, significant heterogeneity was observed between trials (*p* < 0.00001; *I*^2^ = 93%; Fig 2A). In addition to VAS score, Cipriano et al. (2014) reported a significant decrease in the analgesic need (i.e. daily opioid dosage) with an SMD of −2.73 (95% CI, −3.64, −1.82; *p* < 0.00001) [47], indicating a high pain control efficacy of noninvasive SGB.

A subgroup analysis of anticipated optimal dose ranges for noninvasive SGB applied for treating pain revealed that a high dose of US energy (i.e. 3.0 w/cm^2^) resulted in a significant SMD of −0.81 (95% CI, −1.44, −0.18; *p* = 0.01) without heterogeneity between trials (*p* = 0.97; *I*^2^ = 0%). In addition, a high dose of light irradiation (i.e. 150W or > 27J) resulted in a significant SMD of −2.06 (95% CI, −2.66, −1.46; *p* < 0.00001) without heterogeneity between trials (*p* = 0.70; *I*^2^ = 0%; Fig 2B).

### Sympatholytic effects

**Fig 3:**
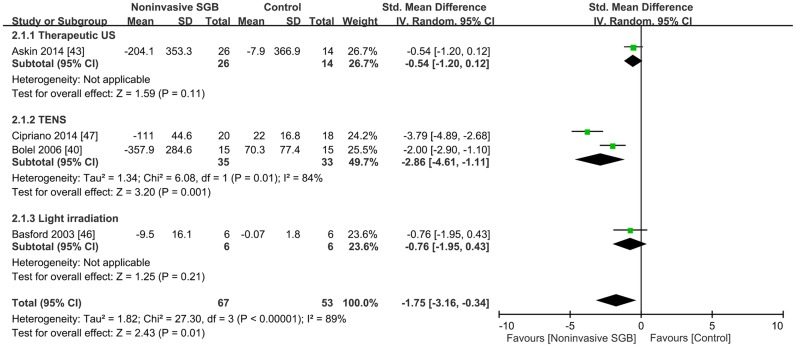
Effect of noninvasive stellate ganglion blockade (SGB) on sympatholytic response compared with that of placebos in four controlled trials grouped according to the type of electrophysical modality used. Trial results plotted on the right-hand side indicate effects favoring noninvasive SGB, and the combined effects are plotted using black diamonds. US = ultrasound; TENS = transcutaneous electrical nerve stimulation.

Immediate sympatholytic responses after noninvasive SGB were determined by measuring sympathetic skin responses in two trials [40, 43], circulating β-endorphin levels in one trial [47], and skin vasomotor reflex in one trial [46]. Because the trials used different measures, the combined results were calculated as SMDs. The combined SMD effect size was −1.75 (95% CI, −3.16, −0.34; *p* = 0.01), and heterogeneity was present (*p* < 0.00001; *I*^2^ = 89%; Fig 3).

### Effect on hemodynamic response

**Fig 4:**
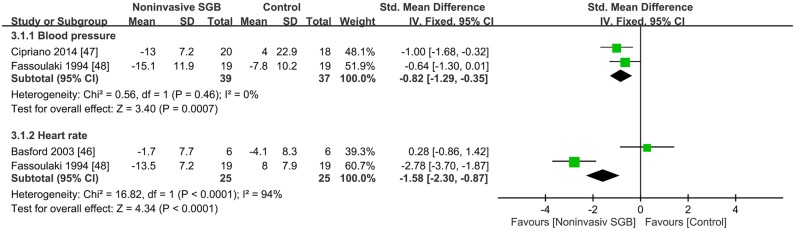
Effect of noninvasive stellate ganglion blockade (SGB) on hemodynamic changes compared with that of placebos. Trial results plotted on the right-hand side indicate effects favoring noninvasive SGB, and the combined effects are plotted using black diamonds.

Immediate hemodynamic responses following noninvasive SGB were determined in three trials [46–48], with two comparing blood pressure and two comparing heart rates (HRs; Fig 4).

Two trials on SGB performed using TENS [47, 48] presented different comparisons of arterial pressure for immediate posttreatment sympathetic responses. The combined SMD effect size was −0.82 (95% CI, −1.29, −0.35; *p* = 0.0007), and heterogeneity was absent (*p* = 0.46; *I*^2^ = 0%; Fig 4).

Two trials, one using TENS [48] and the other using light irradiation [46] for SGB, presented different comparisons of the HR. No significant effect of SGB performed using light irradiation was observed on immediate changes in postirradiation HRs [46]. By contrast, Fassoulaki et al. [48] reported a significant change in postirradiation HRs, favoring the SGB group with an SMD of −2.78 (95% CI, −3.70, −1.87; *p* < 0.0001). The combined SMD effect size was −1.58 (95% CI, −2.30, −0.87; *p* < 0.0001), and heterogeneity was present (*p* < 0.0001; *I*^2^ = 94%; Fig 4).

### Effect on peripheral blood flow

**Fig 5:**
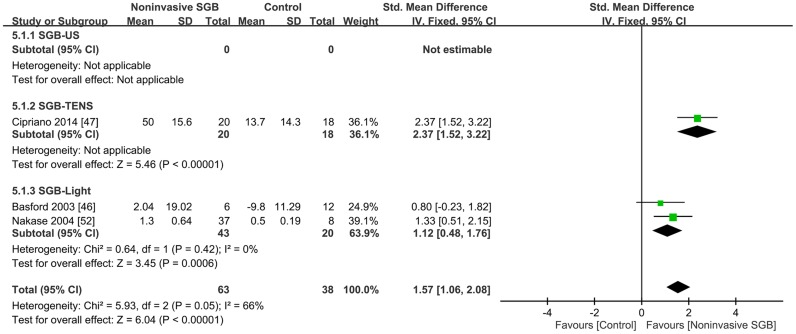
Standard mean difference in peripheral blood flow change between noninvasive stellate ganglion blockade (SGB) and placebo groups in three controlled trials grouped according to the type of electrophysical modality used. Trial results plotted on the right-hand side indicate effects favoring noninvasive SGB, and the combined effects are plotted using black diamonds. US = ultrasound; TENS = transcutaneous electrical nerve stimulation.

Three trials reported continuous data on changes in peripheral blood flow according to different measures [46, 47, 52]. One trial measured femoral blood flow after SGB performed using TENS [47], whereas the other two trials measured tongue blood flow [52] and digital blood flow [46] after SGB performed using light irradiation. The combined analysis revealed that noninvasive SGB significantly increased peripheral blood flow with an SMD of 1.57 (95% CI, 1.06, 2.08; *p* < 0.00001), and heterogeneity was present (*p* = 0.05; *I*^2^ = 66%; Fig 5).

### Effect on peripheral skin temperature

**Fig 6:**
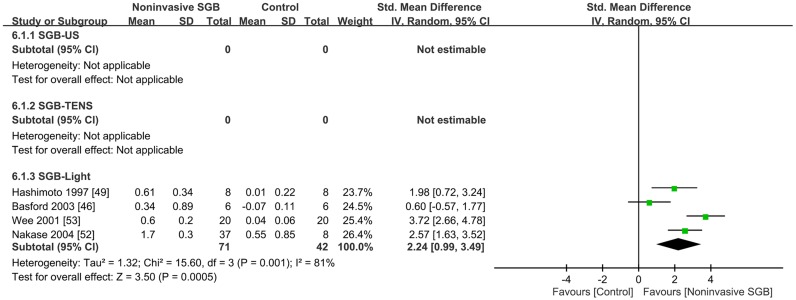
Standard mean difference in the peripheral temperature change between noninvasive stellate ganglion blockade (SGB) and placebo groups in four controlled trials grouped according to the type of electrophysical modality used. Trial results plotted on the right-hand side indicate effects favoring noninvasive SGB, and the combined effects are plotted using black diamonds. US = ultrasound; TENS = transcutaneous electrical nerve stimulation.

Four trials reported continuous data on changes in peripheral skin temperature by using different measures; the methodological quality of two trials was good [46, 49] and that of the other two trials was fair [52, 53]. The combined analysis revealed a significant effect of noninvasive SGB with an SMD of 2.24 (95% CI, 0.99, 3.49; *p* = 0.0005), and heterogeneity was present (*p* = 0.001; *I*^2^ = 81%; Fig 6). An additional trial that was not pooled into the meta-analysis used noninvasive SpO_2_ monitoring in which the signal detection quality was majorly limited because of vasoconstriction and hypothermia in patients with minor trauma [45]. The results indicated that SGB performed using TENS relieved hypothermia, as observed by a reduction in the alarm frequency and time when dropout alerts were initiated, and decreased the difference between the core and skin temperatures.

### Effect on functional mobility and disability

**Fig 7:**
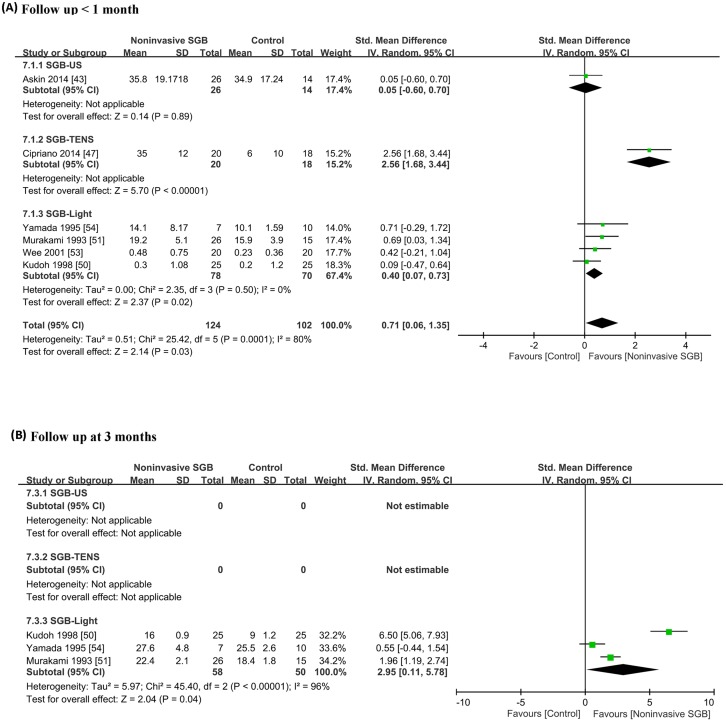
Forest plot of comparisons of outcomes between noninvasive stellate ganglion blockade and placebo groups: (A) short- and (B) medium-term effects on functional mobility and disability outcomes. Trial results plotted on the right-hand side indicate effects favoring noninvasive SGB, and the combined effects are plotted using black diamonds. US = ultrasound; TENS = transcutaneous electrical nerve stimulation.

Six studies provided evidence of short-term improvement in functional mobility or disability following noninvasive SGB treatment. The methodological quality of the three trials was good [43, 47, 50], and that of the other three trials was fair [51, 53, 54] (Fig 7A). Several questionnaire-based and functional outcome measures were used to evaluate disability, functional mobility, and clinical outcomes. One trial [43] evaluated disability of upper extremities after SGB performed using therapeutic US by using the Disability of the Arm, Shoulder, and Hand scale [101]. Three trials [50, 51, 54] examined the paralysis score following SGB performed using light irradiation in patients with facial palsy by using the 40-point and 3-grade Yanagihara scale [102]. One trial [47] evaluated physical function after SGB performed using TENS in patients receiving coronary artery bypass graft surgery by using the 6-min walk test [103]. Another trial [53] assessed the clinical outcome of arm swelling in patients with reflexive sympathetic dystrophy following SGB performed using LPNIR light irradiation by measuring the arm circumference. The combined analysis revealed a significant effect of noninvasive SGB with an SMD of 0.71 (95% CI, 0.06, 1.35; *p* = 0.03), and heterogeneity was present (*p* = 0.0001; *I*^2^ = 80%; Fig 7A).

Only three studies [50, 51, 54] provided evidence for medium-term effects of SGB performed using light irradiation on functional recovery in patients with facial palsy. The combined analysis revealed a significant effect of noninvasive SGB with an SMD of 2.95 (95% CI, 0.11, 5.78; *p* = 0.04; *I*^2^ = 96%, *p* < 0.00001; Fig 7B).

### Side effects of noninvasive SGB

No side effects or adverse events were reported in all included trials. Among the modalities, US, TENS, and LPNIR light irradiation were well tolerated by patients in two [43, 44], four [40, 45, 47, 48], and seven [46, 49–54] trials, respectively.

### Publication bias

**Fig 8:**
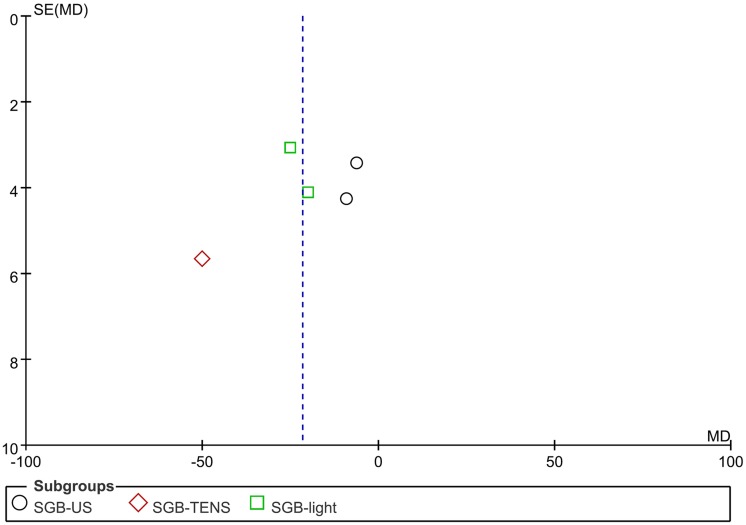
Publication bias plot. Effect size plot for trials with ultrasound (US, circle), transcutaneous electrical nerve stimulation (TENS, diamond), and linear polarized infrared light (square). The effect relative to the placebo is shown on the x-axis, and the standard error is shown on the y-axis. Substantial asymmetry was not observed in the funnel plot of pain reduction through visual inspection. Egger’s linear regression test results indicated no evidence of reporting bias among the studies (*t* = −0.376; *p* = 0.732).

Because only five trials were included in group comparisons for pain reduction, the detection of publication bias from the funnel plot was limited. However, we did not observe substantial asymmetry in the funnel plot of pain reduction through visual inspection (Fig 8). In addition, the results of Egger’s linear regression test provided no evidence of reporting bias among the studies (*t* = −0.376; *p* = 0.732).

## Discussion

In this study, we conducted a comprehensive database search and identified previous controlled and quasi-controlled trials determining the clinical efficacy of noninvasive SGB performed using PAMs in patients with neuropathic pain syndromes or multiple clinical conditions associated with sympathetic hyperactivity. We obtained significant evidence for the efficacy of noninvasive SGB in the short- and medium-term treatment of neuropathic pain.

Noninvasive applications of SGB have been reported to produce effects similar to those of conventional invasive SGB in pain relief, hemodynamic physiology improvement through HR and HRV reduction [56, 69], and increased peripheral blood flow and skin temperature because of vasodilation [35, 60]. Nacitarhan et al. indicated that SGB performed using therapeutic US exerts positive effects on the autonomic nervous system by altering HRV parameters, particularly by reducing the low to high frequency power ratio [42]. Similar results were reported by Yoshida et al. [34]. In this study, we identified significant sympatholytic effects immediately after noninvasive SGB, regardless of the electrophysical modality used. This result indicated that a sympathetic blockade can be effectively performed using noninvasive alternatives to conventional invasive SGB. However, whether the analgesic or sympatholytic effects of noninvasive SGB vary with the type of disease remains unclear. Nevertheless, SGB performed using PAMs is painless and rarely causes side effects. Therefore, it may be a suitable alternative for patients having contraindications for a conventional sympathetic blockade, such as those with a high bleeding tendency.

Because the number of trials identified in our comprehensive search was low, we could not perform a subgroup analysis of optimal dose ranges for noninvasive SGB performed using each electrophysical modality. In addition, we could not compare the application dosage among the four PAMs because different energy forms produce different physiological effects (i.e., therapeutic US generates mechanical vibration energy producing diathermal and nonthermal effects, including cavitation, acoustic streaming, and microstreaming; therapeutic light generates photon energy initiating photobiomodulation effects or athermic photochemical reactions; and therapeutic electricity generates electrical energy inducing electrochemical effects) and energy in different forms penetrates through the skin and into tissues through its specific transdermal pathway of conductance and transformation (i.e., mechanical vibration energy is transdermally conducted through a coupling medium; photon energy is transmitted directly through absorption and indirectly through refraction, dispersion, and reflection; and electric current is delivered by the electric charge flow or by driving charged particles), with varying permeability into deep tissues [39, 104]. However, a high treatment efficacy can be achieved using high doses of energy emitted from the PAMs [105]. Hence, we performed a subgroup analysis of the trials examining different application dosages for noninvasive SGB performed using therapeutic US [43, 44] and light irradiation [49, 52]. Our results demonstrated that compared with a low-power density, the application of high-dose US or light irradiation with a high-power density increased the short-term analgesic efficacy. In addition, a higher analgesic effect was obtained following SGB performed using light irradiation than SGB performed using therapeutic US. Our findings are consistent with those of a previous study regarding the short-term efficacy of electrophysical modality interventions for osteoarthritic knee pain [106].

We observed significant immediate treatment effects and short-term clinical efficacy of noninvasive SGB. However, only three trials [50, 51, 54] investigating the recovery of facial palsy reported medium-term outcomes with a significant effect size. Because the studies reported few results, we could not determine the long-term treatment outcomes over 6 months. Thus, additional studies are required to determine the long-term effect of noninvasive SGB on clinical outcomes.

Sympathetic blockade targeting the stellate ganglion area is believed to be beneficial for patients with a history of chronic musculoskeletal pain syndromes, sympathetically maintained pain syndrome, and clinical conditions associated with vasoconstriction caused by sympathetic hyperactivity. To the best of our knowledge, few systematic reviews or meta-analyses have focused on the clinical efficacy of noninvasive SGB. In this study, we included trials on noninvasive SGB performed using therapeutic US, TENS, LLLT, and LPNIR light irradiation. Our findings support the previous findings of noninvasive SGB performed using PAMs, indicating that phototherapy, TENS, and therapeutic US are beneficial for relieving pain of any etiology. However, although the available results on the efficacy of noninvasive SGB are promising, they demonstrate significant variability. A large-scale prospective randomized controlled trial is required to determine the specific benefits of noninvasive SGB on medium- and long-term outcomes in patients with sympathetic hyperactivity-associated disorders.

Our study has some limitations. First, the articles included in this study were of low methodological quality and had some biases, thus weakening the reliability of the data. Of the nine included studies accurately describing their randomized allocation design [40, 43–50], only five clearly described allocation concealment [40, 43–46]. In addition, of all the 13 included trials, only six [43, 44, 46–48], three [46, 48, 49], and four [43, 44, 46, 48] incorporated subject, therapist, and assessor blinding, respectively. Second, although the data did not suggest substantial publication bias and suggested a significant effect size on pain reduction favoring noninvasive SGB, heterogeneity among the included studies was high. The high heterogeneity may be attributable to the varying designs or low methodological quality of the included studies and low number of studies and participants. Furthermore, only five trials were included for assessing the publication bias of pain reduction and only two studies were available for subgroup comparisons; thus, the results of analyses of publication bias and heterogeneity and the resulting *I*^2^ values were unreliable. Third, although we performed a meta-analysis of all the included trials using SGB with different PAMs, a subgroup analysis of different PAM types could not be performed because the number of articles included for each electrophysical modality was low and the measurement tools used to assess clinical outcomes varied among trials. Nevertheless, all the studies included in the statistical analysis of the analgesic effect [43, 44, 47, 49, 52] reported pain outcomes by using VAS scores, enhancing the ease of comparing treatment efficacies among different PAM types for noninvasive SGB and increasing the generalizability of our results to neuropathic pain of different etiologies [107]. Finally, because of limited published evidence, we could identify only the immediate treatment effects (within 1 day) and short-term outcomes (up to 1 month) after noninvasive SGB application. Additional studies on noninvasive SGB performed using the PAMs discussed in this study are required to determine whether the sympatholytic effects are beneficial in long-term clinical outcomes.

## Conclusions

The results of this study demonstrated that noninvasive SGB performed using PAMs relieved pain and improved autonomic dysfunction in patients with sympathetic hyperactivity disorders. The results indicate that sympathetic blockade can be effectively performed with few side effects by using noninvasive SGB with PAMs. Our findings can assist clinicians in making decisions regarding alternatives to conventional SGB and selecting the optimal treatment strategy. However, additional high-quality, large-scale, randomized controlled trials with long-term follow-up are required to further establish the efficacy of PAMs in noninvasive SGB for pain management.

## Supporting Information

S1 PRISMA Checklist PRISMA 2009 checklist. (DOC)
